# Replication of H9 influenza viruses in the human *ex vivo* respiratory tract, and the influence of neuraminidase on virus release

**DOI:** 10.1038/s41598-017-05853-5

**Published:** 2017-07-24

**Authors:** Renee W. Y. Chan, Louisa L. Y. Chan, Chris K. P. Mok, Jimmy Lai, Kin P. Tao, Adebimpe Obadan, Michael C. W. Chan, Daniel R. Perez, J. S. Malik Peiris, John M. Nicholls

**Affiliations:** 1 School of Public Health, LKS Faculty of Medicine, The University of Hong Kong, Pokfulam, Hong Kong, SAR China; 2 HKU-Pasteur Research Pole, The University of Hong Kong, Hong Kong, SAR China; 3 Department of Population Health Poultry Diagnostic and Research Center, College of Veterinary Medicine University of Georgia, Athens, USA; 4 Department of Pathology, The University of Hong Kong, Queen Mary Hospital, Pokfulam, Hong Kong, SAR China; 5 Department of Paediatrics, Faculty of Medicine, The Chinese University of Hong Kong, Hong Kong, SAR China

## Abstract

H9N2 viruses are the most widespread influenza viruses in poultry in Asia. We evaluated the infection and tropism of human and avian H9 influenza virus in the human respiratory tract using *ex vivo* respiratory organ culture. H9 viruses infected the upper and lower respiratory tract and the majority of H9 viruses had a decreased ability to release virus from the bronchus rather than the lung. This may be attributed to a weak neuraminidase (NA) cleavage of carbon-6-linked sialic acid (Sia) rather than carbon-3-linked Sia. The modified cleavage of N-acetlylneuraminic acid (Neu5Ac) and N-glycolylneuraminic acid (Neu5Gc) by NA in H9 virus replication was observed by reverse genetics, and recombinant H9N2 viruses with amino acids (38KQ) deleted in the NA stalk, and changing the amino acid at position 431 from Proline-to-Lysine. Using recombinant H9 viruses previously evaluated in the ferret, we found that viruses which replicated well in the ferret did not replicate to the same extent in the human *ex vivo* cultures. The existing risk assessment models for H9N2 viruses in ferrets may not always have a strong correlation with the replication in the human upper respiratory tract. The inclusion of the human *ex vivo* cultures would further strengthen the future risk-assessment strategies.

## Introduction

Most influenza viruses are widely established in the waterfowl population. While much attention has been given to the H5N1 and H7N9 subtypes, because of their disease severity in humans, another subtype, H9N2, has widely circulated in poultry in many regions of the world. H9N2 was first detected in turkeys in 1966, shorebirds in 1988, and then in ducks and chickens^1, 2^. It is now the most widespread and prevalent influenza virus in Asia. The government of Hong Kong Special Administrative Region implemented interventions starting from 2001 to control this infection, by executing a mandatory monthly rest day, live quail sale ban, and in the subsequent years a biweekly rest day in the wet markets. Prior to these interventions in live bird market trading, the H9N2 virus isolation rate in Hong Kong’s live poultry markets was around 5% of all birds tested increasing to almost 20% in game-birds such as quail^3^. These isolation rates are presumably similar in the greater China region in which H9N2 is regarded as the most prevalent subtype of influenza in chickens (reviewed in ref. 4), where market interventions applied in Hong Kong have not been used. Phylogenetic analysis has shown that the three main lineages (Korean, G1 and Chicken/Beijing/1/94-like) of H9N2 viruses have developed from Ck/Bei and reassorted, with other H9 strains^1^.

Even though H9N2 infection is mainly confined to poultry, H9N2 viruses have been detected repeatedly in swine in China^5, 6^. Laboratory confirmed cases of H9N2 infection in humans have been reported in 1999^7, 8^, 2005^9^, 2008^10^ and 2013^11^ and it appears that immunocompromised individuals may be more susceptible to H9N2 infection^10^. The early human H9N2 cases were identified in Hong Kong from 1999 to 2003^9, 12^ (reviewed in ref. 10), in children who presented with a mild influenza-like illness. Later cases identified in Hong Kong^10^ and Mainland China^11^ occurred in adults. These isolated cases might only reflect the tip of the iceberg, as the high prevalence of anti-H9 antibodies among poultry workers in China^13^, Vietnam^14^, and Iran^15^ raises the possibility that mild or asymptomatic infection of H9N2 in human could be common.

Serological surveillance in China has shown that 1.4% of the population have antibodies to H9N2 as determined by haemagglutination inhibition and virus neutralization^16^. Among pig farm workers in the Southern China, 1.4% of staff were seropositive with 4% of pigs showing seroconversion^17^. A more detailed sero-epidemiological study from Thailand found that 4.7% of the population had elevated antibody titres to H9N2^18^. These previous publications thus indicate that H9 infections in human could be common in the Asian-Pacific region.

Recent publications have sought to risk assess the pandemic potential of H9N2 viruses using *in vitro* cell culture and laboratory animal infection systems^19^. For many years the ferret has been used as a surrogate human model for influenza studies reviewed in (Belser *et al*.)^20^ and there has been limited data on replication competence in the *ex vivo* respiratory organ culture of ferret with good validation. We sought to explore the tissue tropism of the wild type H9 viruses isolated from poultry and humans in the *ex vivo* cultures of the human bronchus and lung and to investigate if there was any unique viral genomic signature which would determine successful replication in the human airways and which would act as a predictor, or correlate of infection in humans. We also sought to explore the how previously characterized changes in the internal viral proteins would affect replication as well comparing the N1 against the N2 for replication competence. As changes in amino acid at position 190 in haemagglutinin (HA) have been proposed to affect receptor binding^21^ and serial passage in animal models has also been demonstrated to affect replication^22^. We produced a recombinant virus with this change with 464419/H9N2 as the﻿ backbone (listed in Table 1 and Supplementary materials, Table S1) in our assessment in *ex vivo* human respiratory tissue cultures.

**Table 1:** List of viruses used and discussed in this study, including their abbreviation.

Strain name	Subtype	Abbreviation	
A/Shanghai/2/2013	H7N9	Sh2	WT
A/Quail/Hong Kong/G1/1997	H9N2	G1	WT
A/Duck/Hong Kong/Y280/1997	H9N2	Y280	WT
A/Duck/Shantou/2030/2001	H9N1	Dk/2030	WT
A/Chicken/Hong Kong/SSP117W/2009	H9N2	Ck/SSP	WT
A/Chicken/Hong Kong/YU341/2008	H9N2	Ck/YU341	WT
A/Chicken/Hong Kong/NT449/2007	H9N2	Ck/NT449	WT
A/Hong Kong/1073/1999	H9N2	1073	WT
A/Hong Kong/2108/2003	H9N2	2108	WT
A/Hong Kong/226995/2008	H9N2	226995	WT
A/Hong Kong/464419/2009	H9N2	464419	WT
A/Hong Kong/464419/2009 HA-D190E	H9N2	D190E	RG
A/California/04/2009	H1N1pdm	Ca04	WT
G1 HANA: 6 × A/California/04/2009 internal genes	H9N2	rgH9N2	RG
G1 HA: 7 × A/California/04/2009 genes	H9N1	rgH9N1	RG
A/guinea fowl/Hong Kong/WF10/1999	H9N2	WF10	WT
A/Memphis/14/1998	H3N2	M98	WT
A/Netherlands/602/2009	H1N1pdm	/	WT
WF10 HA: 7 × A/Netherlands/602/2009 genes	H9N1	1WF10	RG
WF10 HANA: 6 × A/Netherlands/602/2009 internal genes	H9N2	2WF10	RG
WF10 HANA: 6 × M98 internal genes	H9N2	2WF10:6M98	RG
A/Ferret/Maryland/P10_UMD/2008	H9N2	P10	AD
P10 HA: 7 × A/Netherlands/602/2009 genes	H9N1	1P10	RG
P10 HANA: 6 × A/Netherlands/602/2009 internal genes	H9N2	2P10	RG
P10 HANA: 6 × WF10 internal genes	H9N2	2P10:6WF10	RG

Key: WT, wild type; RG, reverse genetic constructed; AD, ferret-adapted.

## Results

### Human H9N2 influenza virus replication in the human bronchus and lung organ cultures

**Figure 1:**
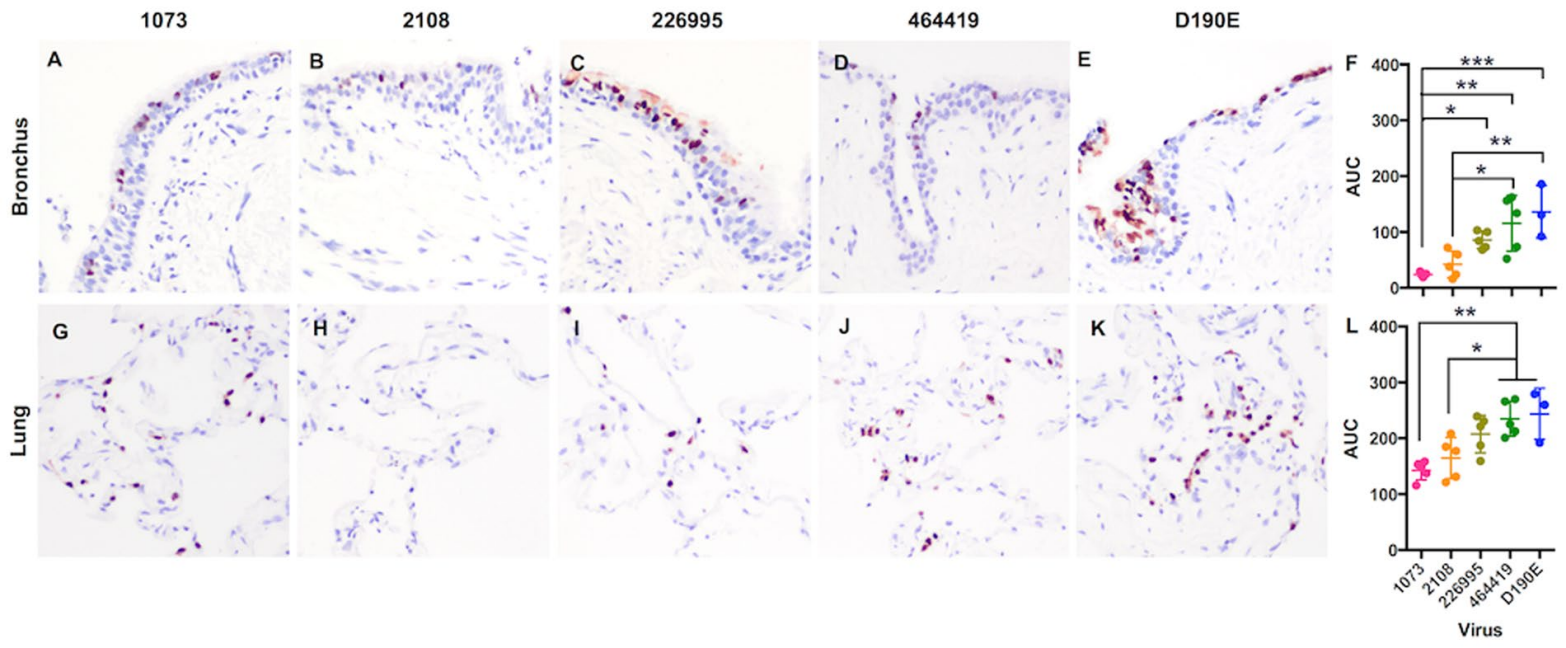
The cellular distribution of influenza nucleoprotein in the human bronchus and lung after the infection of human H9N2 viruses. The cellular localization of viral antigen (reddish brown) in (**A** to **E**) bronchi, and (**G** to **K**) lung infected with (**A** and **G**) A/HK/1073/99 (H9N2), (**B** and **H**) A/HK/2108/03 (H9N2), (**C** and **I**) A/HK/226995/08 (H9N2), (**D** and **J**) A/HK/464419/09 (H9N2) viruses and (**E** and **K**) A/HK/464419/09-D190E (H9N2) at 24hpi were shown. The chart showed the mean and the SD of the AUC values of each virus pooled from at least three independent experiments of each virus (**F**) in the bronchus and (**L**) in the lung cultures were shown. *p < 0.05; **p < 0.01; ***p < 0.005.

We have determined the infectivity﻿ ﻿and viral replication competence of human H9N2 influenza viruses A/Hong Kong/1073/1999(1073), A/Hong Kong/2108/2003 (2108), A/Hong Kong/226995/2008 (226995) and A/Hong Kong/464419/2009 (464419) (Table 1) in *ex vivo* cultures of human bronchus and lung. Immunohistochemistry studies indicated that there was infection of the bronchial epithelium (Fig. 1A–E), and the lung (Fig. 1G–K). The lung *ex vivo* cultures infected with human H9N2 viruses, 1073 and 464419 showed a greater extent of infection than other two H9N2 strains, 2108 and 226995 (Fig. 1G and J). Virus replication kinetic data indicated there was no significant increase in the viral titer in the bronchus with 1073, 2108 and D190E, over the infection time course (Supplementary materials, Figure S1A,B and E). Since the thermal inactivation curves of these H9 viruses showed a drop of infectious virus titer  to undetectable levels by 24 hpi (Supplementary materials, Figure S2), the extent of the overall viral replication was assessed with the area-under-curve (AUC) analysis using a trapezoid rule. The AUCs calculated from 24 to 48 hpi above the detection limit of each virus were compared (Fig. 1F and L). In the bro﻿nchial﻿ (Fig. 1F) and lung cultures (Fig. 1L),﻿ 1073 (pink dots) and 2108 (orange dots) showed significantly less replication when compared 464419 and its mutant D190E. However, there was no differences between the 464419 (green dots) with and without the HA-D190E (blue dots) mutation in both the bronchus and the lung.

### Avian H9 influenza virus replication in human bronchus and lung organ cultures

**Figure 2:**
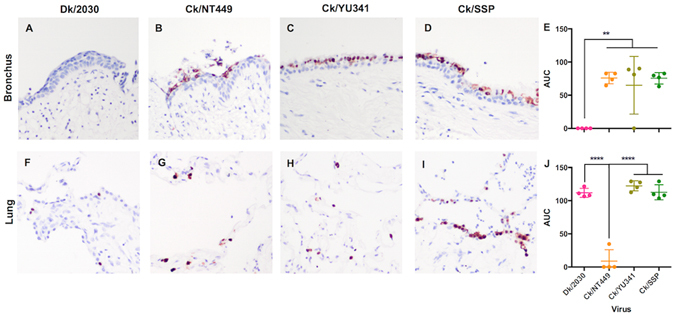
The cellular distribution of influenza nucleoprotein and the viral replication extensiveness in the human bronchus and lung after the infection with avian H9 viruses. The cellular localization of viral antigen in (**A** to **D**) bronchi, and (**F** to **I**) lung infected with avian (**A** and **F**) A/Duck/ST/2030/2001 (H9N1), (**B** and **G**) A/Chicken/HK/NT449/2007 (H9N2), (**C** and **H**) A/Chicken/HK/YU341/2008 (H9N2) and (**D** and **I**) A/Chicken/HK/SSP117W/2009 (H9N2) viruses at 24hpi were shown. The chart showed the mean and the SD of the AUC values of each virus pooled from at least three independent experiments (**E**) in the bronchus and (**J**) in the lung cultures were shown. **p < 0.01, ****p < 0.001.

In the bronchus, Dk/2030 (H9N1) did not infect or replicate in the human bronchial tissue (Fig. 2A), in contrast to the three H9N2 avian viruses which infected (Fig. 2B to E) and replicated extensively (Fig. 2E). Nevertheless, H9N1 had a lung tropism and replicated equally well when compared to Ck/YU341 and Ck/SSP (Fig. 2J). In contrast, the *ex vivo* cultures of human lung replication competence of Ck/NT449 was significantly lower than other H9 viruses (Fig. 2J) though positive antigen was identified (Fig. 2G).

### H9 virus replication in human bronchus and lung organ cultures using viruses previously demonstrating replication competence in the ferret

In order to determine if the difference in replication in the bronchus was due to the functional balance between the surface HA and NA, or driven by the virus internal gene cassette, we examined the virus tropism using eight strains of H9 virus which had previously shown different replication abilities in the ferret: WF10 (an avian H9N2) and its recombinant variants (1WF10, 2WF10, 2WF10:6M98), a ferret-adapted variant (P10), and it variants (1P10, 2P10, 2P10:6WF10).

**Figure 3:**
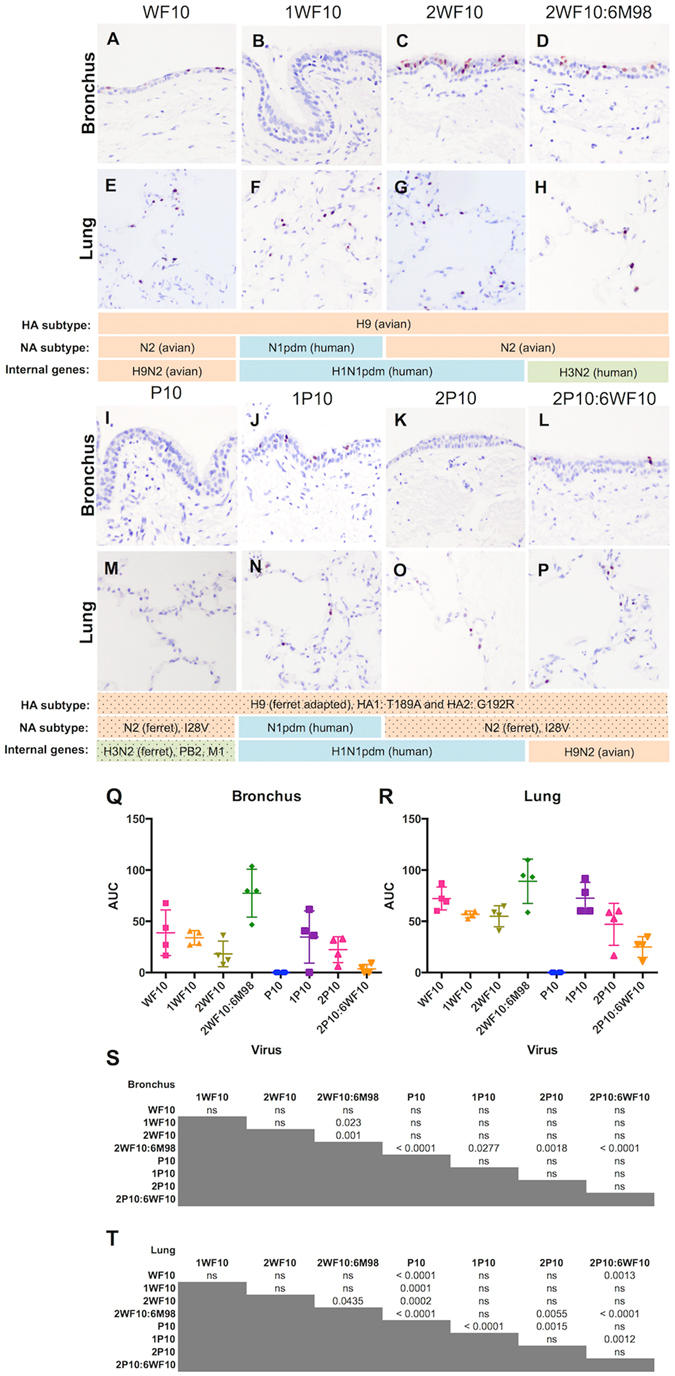
Cellular distribution influenza nucleoprotein in the human bronchus and lung after the infection with recombinant and ferret-adapted H9 viruses and the comparison of their replication competence. Cellular localization of viral antigen in (**A** to **D**) and (**I** to **L**) bronchi, and (**E** to **H**) and (**M** to **P**) lung infected with reassortant viruses generated using H9N2 and H1N1pdm (WF10, 1WF10, 2WF10), and using H9N2 and H3N2 (2WF10:6M98). (**I** and **M**) P10 was the ferret-adapted virus harvested after 10 serial passages in ferrets of 2WF10:6M98, with mutation sites at HA1: T189A, HA2: G192R, NA: I28V, PB2: L374I, M1: H110Y, at 24hpi. Virus replication competence (**Q**) in the human bronchus and (**R**) in the human lung were expressed in AUC as described in the method. The chart showed the mean and the SD of the AUC pooled from at least four independent experiments with the result tables (**S** and **T**) showing the statistical comparison between viruses, *p < 0.05; **p < 0.01, ***p < 0.005, ****p < 0.001 and ns: *p* > 0.05.

The wild type avian H9N2 virus, WF10, infected the human bronchus and lung (Fig. 3A and E). In addition, the replacement of NA and the internal genes from H1N1pdm (1WF10) or the replacement of its internal gene cassette by H1N1pdm (2WF10), did not statistically alter the replication competence of the virus in human bronchus and lung compared to the WF10 (Fig. 3S and T). The more extensive infection observed in 2WF10 infected bronchial tissues (Fig. 3C) could be contributed by the balance of the HA and NA in 2WF10 rather than the avian-HA and the human-NA of the 1WF10. Strikingly, the internal gene cassette of human H3N2 (Fig. 3D and H, 2WF10:6M98) significantly increased the replication competence of the virus in the bronchus (Fig. 3Q and S, green dots) when compared to 1WF10 (*p* = 0.023) and 2WF10 (*p* = 0.001).

The P10 virus was derived by serial passage of 2WF10:6M98 in ferrets for ten times^22, 23^. In contrast to the ferret model studied, P10 did not appear to infect or replicate in the human bronchus or the lung (Fig. 3Q and R) and immunohistochemistry was negative (Fig. 3I and M). Furthermore, it can be seen that comparing 2WF10:6M98 (Fig. 3D and H), the ferret adapted P10 has a compromised replication competence in the human bronchus and the lung. Moreover, the mutations of P10 in HA and NA acquired would contribute to the less competence phenotype, with the fact that 2P10:6WF10 replicated less in the human bronchus (*p* = 0.14) and in the lung (*p* = 0.0013) when compared to WF10. The mutated surface proteins were less fit to replicate in the human respiratory tract. Nevertheless, the internal gene cassette of H1N1pdm in 1P10 and the 2P10 partially rescued their replication in the human bronchus (Fig. 3Q, though not reaching statistical significance) and in the human lung (Fig. 3T, *p* < 0.0001 and *p* = 0.0015, respectively).

### Investigation of the role of NA in determining virus replication

**Figure 4:**
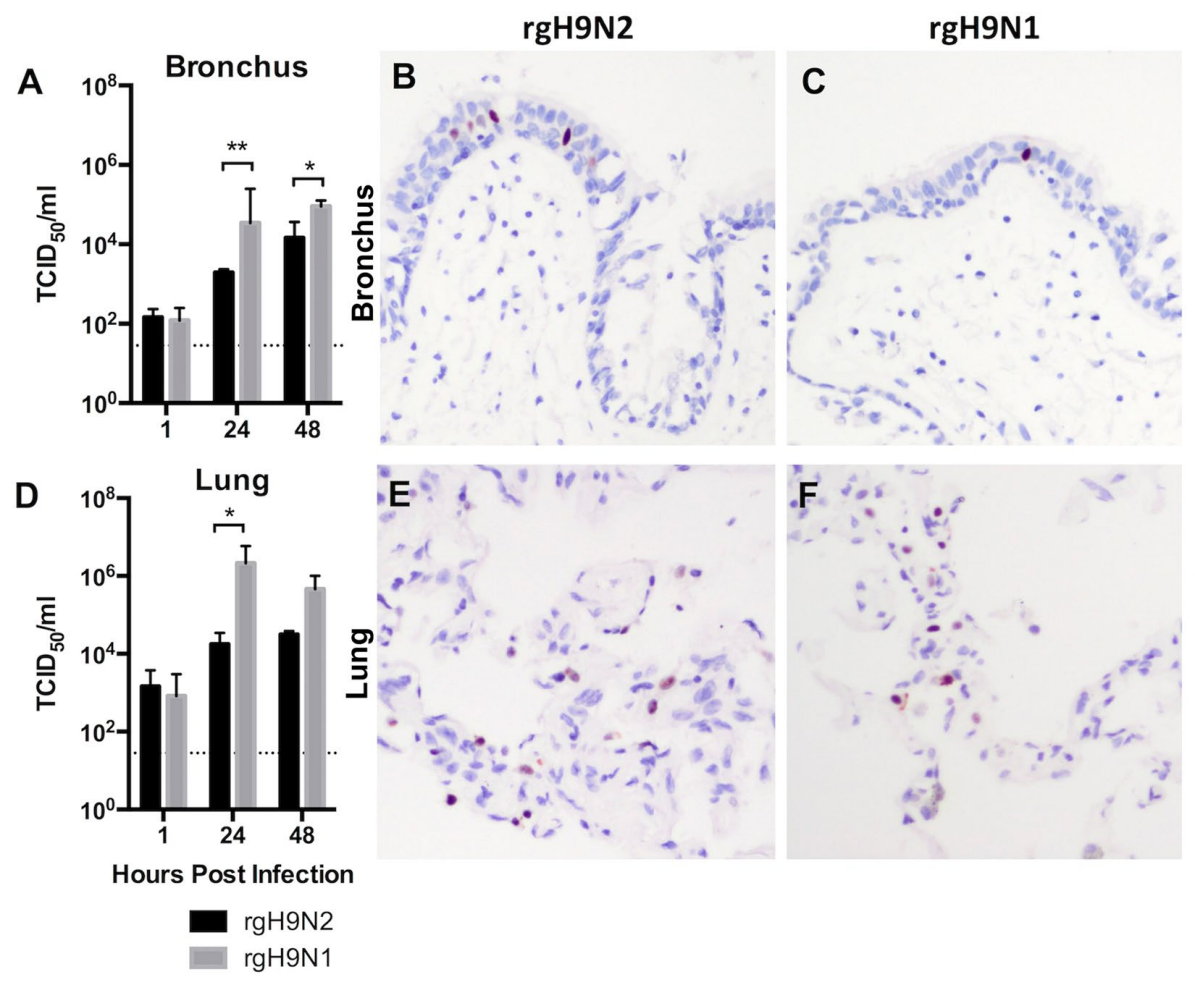
Influence of neuraminidase on H9 virus replication. Replication in the (**A**–**C**) bronchus and (**D**–**F**) lung of reverse genetics H9N2 containing the HA and NA of H9N2/G1 and the internal genes from a pandemic H1N1 virus (A/California/04/2009) (rgH9N2), and a comparing virus with only HA of H9N2/G1 and the other seven genes from (A/California/04/2009) (rgH9N1). The chart showed the mean and the SEM of the virus titer pooled from at least three independent experiments. Asterisks indicated statistical significant increase in viral yield at the same time point between the two virus using paired student *t*-test. *p < 0.05; **p < 0.005.

To further investigate the correlation between the results in human *ex vivo* organ culture and animal study, another set of recombinant viruses was used. The first H9 virus, rgH9N2, had the HA and NA proteins derived from G1 (H9N2) and its internal genes from the A/California/04/2009 (H1N1pdm). The second H9 virus, rgH9N1, had the HA protein derived from G1 and the seven other genes from Ca04. In our human *ex vivo* bronchial and lung organ cultures, both viruses showed the ability to replicate over time (Fig. 4A and D), and the rgH9N1, which contained the human pandemic-NA, replicated better than rgH9N2 in cells derived from both anatomical sites.

### Neuraminidase cleavage of sialoglycans

**Figure 5:**
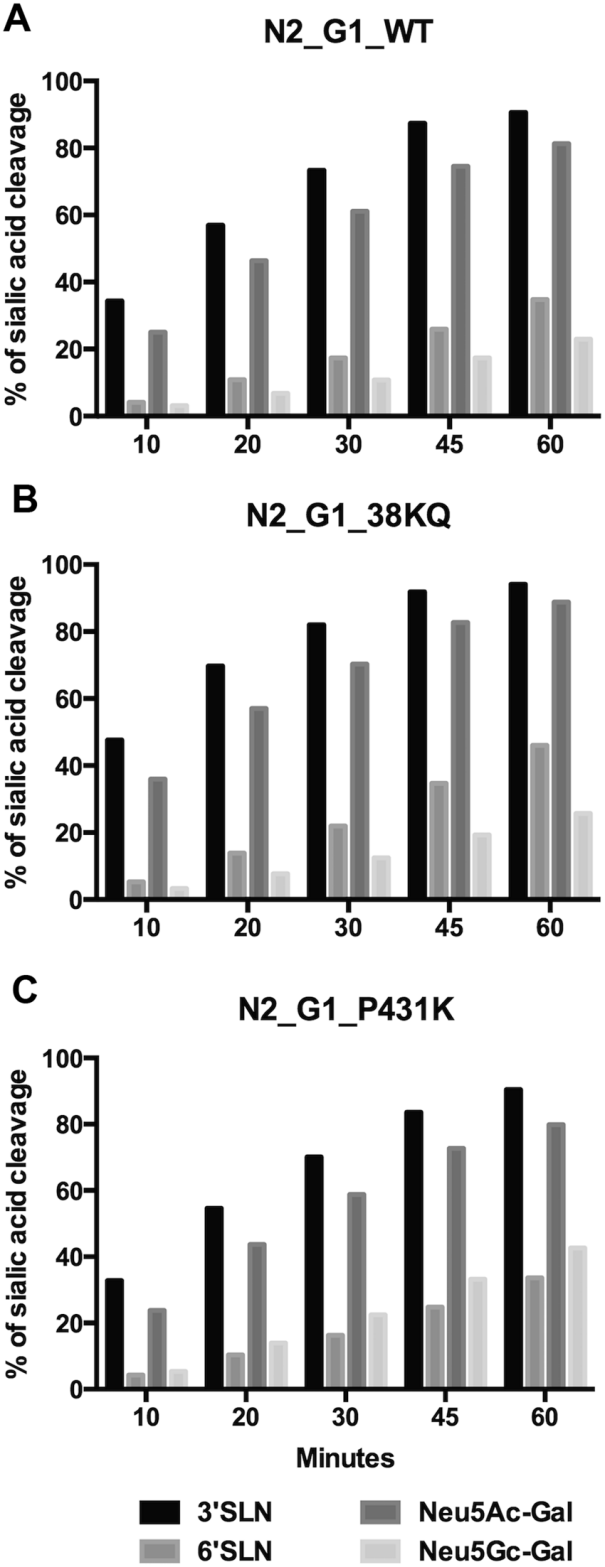
Cleavage of glycans by influenza neuraminidase. Cleavage of different sialylated glycans 3-sialylolactosamine (3-SLN), 6-sialylolactosamine (6-SLN), Neu5Ac-Galactose (Neu5Ac-Gal) and Neu5Gc-Gal by (**A**) H9N2 GI, (**B**) H9N2 with replacement of the two amino acid stalk deletion and (**C**) H9N2 with mutation of P431K.

As there was a difference in replication between the rgH9N1 and rgH9N2, we investigated what role the NA contributed to the efficient virus budding in the human airways. The early H9N2 viruses that infected humans contained a deletion of two amino acids (38 and 39) in the stalk region (similar to G1), but also contained similar glycosylation sites with other N2s^12^. NMR spectroscopy was used to assess NA activity of the wild type G1 virus (N2_G1_WT), a reverse genetics virus where the two amino acid deletions were restored (N2_G1_38KQ) and a reverse genetics virus with a mutation in P431K which this substitution could change the orientation of the NA 430 loop and influence Sia binding^24^. The main role of viral NA is to cleave Sia from the epithelial surface as well as facilitate viral penetration through the sialylated mucus layer. While all Sia in humans is primarily N-acetylatyed (Neu5Ac), certain animal species such as pigs have a high degree of N-glycolylneuraminic acid (Neu5Gc). High throughput studies by Li and colleagues have demonstrated that NA activity is more efficient in cleaving 2–3 linked Sia than 2–6 linked Sia, with human viruses also efficient in using Neu5Gc as a substrate^25^. Since certain amino acid changes in NA may lead to a shift in cleavage preference from Neu5Ac or Neu5Gc, and thus affect tissue tropism, we produced recombinant viruses that contained these signature amino acid changes and examined the NA activity using 3 substrates that contained Neu5Ac – 3 sialyllactosamine (3SLN), 6–sialyllactosamine (6-SLN) and Neu5Ac-Gal, and a substrate containing Neu5Gc (Neu5Gc-Gal). In all three viruses studied, there was a greater cleavage of the 3-linked Sia than the carbon 6-linked Sia (Fig. 5A–C). Supplementary materials, Table S1 shows that only four of the H9N2 viruses had this stalk deletion (G1, Y280, 1073 and P10) by amino acid sequences comparison. However this did not appear to have any effect on replication in the *ex vivo* culture of human bronchus or lung tissues by comparing the viral replication competence of H9N2 viruses with residues 38–39 deletion in NA stalk, 1073 and P10 with other viruses without this deletion such as 2108, 226995 and 464419 (Fig. 1F and L) and did not affect the preference between Sia α2–6 glycan or Sia α2–3 glycan cleavages (Fig. 5B), although the overall enzymatic activities were slightly higher with the 38KQ insertion in NA.

## Discussion

One of the factors determining successful replication of influenza viruses in the human respiratory tract involves the interplay between the binding affinity, determined by the host glycan-virus haemagglutinin interaction, and the release, determined by the activity and specific of the viral NA. In order to assess whether there was a difference in the replication abilities of different H9 viruses, we utilized a previously established *ex vivo* culture system to investigate the replication ability and tropism of H9 viruses. We found that apart from the P10 virus from ferrets, all H9 viruses were able to infect the *ex vivo* bronchus and lung with greater replication identified in the lung than the bronchus. This overall lower replication ability in the bronchus may be due to a loss of balance between HA and NA, so that even though virus is being produced, the activity of the NA prevents efficient virus release. Indeed, electron microscopy on two of the bronchial samples showed increased numbers of viral particles on the surface of the epithelium (Supplementary materials, Figure S3).

In previous publications using *ex vivo* cultures of the human respiratory tract we have used increase in viral titer expressed in TCID_50_/ml as evidence of successful replication. Our studies have shown that in the absence of permissive cells to support viral replication, thermal inactivation leads to the loss of viral infectious titer by 24 hours (Supplementary materials, Figure S2), Therefore, in this study, we also used AUC between 24–48 hpi to express the overall replication competence of the virus. This evaluation correlated well with the positive antigen detection in the immunohistochemical stained tissues and the viral titer (Supplementary materials, Figure S1).

The role of the internal genes in determining H9 virus transmission in ferrets has been extensively studied by the Perez and other groups^22, 26–30^. When the H9 had internal genes derived from H3N2 (e.g. 2WF10:6M98), there was an increased replication and expanded tissue tropism compared to the wild type, together with a gain in transmission by direct contact in ferrets, but aerosol transmission was only possible only after repeated serial passage in ferrets and the acquisition of further adaptive mutations^27^. When the H3N2 internal genes were replaced with a triple reassortant internal gene (TRIG) cassette, respiratory transmission was possible in ferrets leading to clinical disease. If the internal genes were from pH1N1 in mice this also led to documented clinical disease.

Many of the H9N2 viruses have the capacity to bind both Siaα2–3 Gal “avian” and Siaα2–6 Gal “human” terminated receptors (found in the human upper and lower respiratory tract)^23^, however binding was predominantly to Siaα2–6 Gal glycans. Amino acid changes in the HA have been shown to determine the cell tropism by altering this α2–3 or α2–6 binding affinity^27^, and HA-Q226L of avian H9N2 viruses plays a key role on this switching^23^. Sequence analysis of the viruses used in our study (Supplementary materials, Table S1) shows that only the Dk/2030, Ck/Yu341, and 464419 (H9N2) maintained the Q226 but were not similar in the distribution of viral antigen and replication in the bronchus and lung. Wan and colleagues in 2008 demonstrated that the amino acid leucine (L) at 226 was the crucial determinant for direct transmission of avian H9N2 in ferrets with more replication in the upper respiratory tract and multi organ dissemination^23^. They also showed aerosol transmission in ferrets when the H9N2 avian-human reassortant (2WF10:6M98) that caused direct transmission between ferrets was serially passaged in ferrets, leading to the acquisition of additional amino acid changes in the HA^27^. Recently, a HA-E190D mutation (the HA 190D is usually found in human seasonal influenza viruses) is observed in a human H9N2 virus (A/Hong Kong/464419/2009), but this virus still contained the HA-226Q that maintained preferential binding to Siaα2–3 rather than Siaα2–6^10^. The D190E substitution appeared to have no effect on replication as assessed by AUC. One of the human isolates had a D190V change (2108) and this appeared to have the lowest replication in the lung tissues.

In our *ex vivo* model, the main significant changes impacting on the replication in the human bronchus were the change of the internal genes from H1N1 to H3N2 (2WF10 and 2WF10:6M98) (Fig. 3C,D), and the serial passage of 2WF10:6M98 to P10 (Fig. 3). In contrast to the ferret data, the serially passaged virus (P10) had a limited ability to replicate in the bronchus (Fig. 3S), even when the “favourable” H3N2 internal genes were present. This finding indicates that the viruses with airborne transmission in the ferret model might not always have efficient replication competence in the human bronchus and thus extrapolation of these data to virus transmissibility between humans may not be straightforward. The signature amino acid changes seen after serial passage were not favourable to replication in the bronchus or lung but that these could be improved by replacing the N2 (ferret) with N1 (human) in the lung (Fig. 3M,N,S and T).

Previously we did not know whether the lack of human-to-human transmission of H9N2 was due to poor binding because of HA difference, replication competence because of the internal gene cassette or virus release due to different N1 or N2 proteins. As some *ex vivo* infections had positive IHC but low virus replication, this suggests that a low virus release may be a more likely scenario. We have demonstrated that replication in the bronchus and the lung differs between the rgH9N1 and rgH9N2 (Fig. 4), and that in the serially passaged virus 1P10 has greater replication than P10 (Fig. 3T). Thus it is possible that one of the main reasons for the poor transmission between humans of H9N2 would be the N2 is not well-adapted or “fit” for the human airway. It has previously been reported that there is a difference in cleavage of substrates between the N1 and N2 with the latter showing a lower substrate specificity than N1^31^. Our NMR and N2 mutation assay confirmed that 2–6 linked Sia (which comprised most of the mucin of the upper respiratory tract)^32^ was less efficiently cleaved than 2–3 linked Sia (Fig. 5A–C).

The N2 of H9N2 shows some degree of amino acid variation between strains. The early H9N2 viruses that infected humans contained a deletion of two amino acids (38 and 39) in the stalk region (similar to G1), but also contained similar glycosylation sites with other N2s^12^. In contrast, Y280 contained a deletion of three amino acids (position 63–65), however many other H9N2 isolates (including those that were isolated from the 2008 and 2009 human H9N2 infections) have no deletions^33^. This stalk deletion has been associated with an adaptation of viruses from waterfowl to terrestrial poultry, and was present in the emergence of the H7N9 virus that infected humans in 2013^34^. This deletion increased replication in mice and chickens with increased severity of primary disease^35^, though it was not the case in the *ex vivo* infection of human respiratory tissue by 1073 and P10 in this study. Furthermore, a recent study on the amino acid changes in NA resulting in airborne transmission in chickens^36^ showed that none of the amino acid changes in the airborne transmissible and non-airborne transmissible virus involved the stalk. Similar to a previous publication^37^, the mutation of the P431K increased cleavage of Neu5Gc (Fig. 5C).

## Conclusions

An important aspect in the study of influenza virus biology and ecology has been whether the airborne transmission of an influenza virus in ferrets can be directly and universally applied to imply transmissibility between humans, and whether the sites of replication of these reassortant H9N2 viruses in ferrets may comparable when the human respiratory tract is used. This consideration is important, as replication of H9N2 in the ferret has been included as a risk assessment of H9N2 viruses^19^.

The risk assessment models published previously placed emphasis on replication in ferrets, pigs and the role of HA226 but we found that this was not always a reliable predictor of an increased replication competence in *ex vivo* cultures of the human respiratory tract. Since N2 is a relatively weak NA and the bronchial environment has abundant 2–6 linked glycans, we now propose that the action of the NA as well as *ex vivo* cultures of the human bronchus and lung should be considered in conducting future risk assessments of H9N2 viruses.

Our *ex vivo* studies have shown the human adult bronchus and lung is readily susceptible to infection by H9N1 and H9N2 viruses that are commonly found in poultry in the region, with a greater degree of replication identified in the lungs than bronchi. We found that amino acid changes favouring replication and airborne transmission identified in the ferret model did not always lead to a better replication in the human system. *Ex vivo* cultures of the human lung and bronchus provides a useful system for investigating avian-to-human and human-to-human transmission as the two important questions regarding the potential zoonotic and pandemic threat posed by H9N2 viruses.

## Materials and Methods

### Virus culture

Four wild type strains of avian H9 influenza virus and four wild type strains of human H9N2 virus were employed in this study and detailed in Table 1. The signature amino acid changes are listed in Supplementary materials, Table S1. A reverse genetics virus 464419-D190E re-constructed with A/Hong Kong/464419/2009 (H9N2) backbone was prepared as described^38^. All viruses were plaque purified and passaged three times in Madin-Darby Canine Kidney (MDCK) cells and their identity was confirmed by sequencing.

In order to study whether H9 viruses that replicated in the ferret model would replicate in our *ex vivo* system, a panel of eight viruses previously generated using plasmid based reverse genetics and tested in ferret models were used. Briefly, these viruses include the parent virus A/guinea fowl/Hong Kong/WF10/1999 (H9N2, WF10), and three H9N2 avian-human reassortant viruses: 1WF10, which contains the HA gene of WF10 and the NA and the six internal genes of A/Netherlands/602/2009 (H1N1pdm); 2WF10, which contains the HA and NA genes of WF10 and the six internal genes of H1N1pdm, and 2WF10:6M98 which contains the HA and NA genes of WF10 and the six internal genes of A/Memphis/14/1998 (H3N2, M98).

The second set of viruses was based on A/ferret/Maryland/P10_UMD/2008 (P10) (H9N2), which was the result of 10 serial passages in ferrets of the 2WF10:6M98 (avian-human H9N2:H3N2)^22^. Compared with the parent virus, P10 possessed mutations at surface proteins, HA1-T189A, HA2-G192R and NA-I28V, internal proteins, PB2-L374I and M1-H110Y. In addition, three reassortant viruses with the surface protein(s) of P10 were generated. 1P10 contains the HA from P10 and the other even genes from H1N1pdm, whereas 2P10 contains the HA and NA genes from P10 and the six internal genes from H1N1pdm. Lastly, the 2P10:6WF10 which encodes the HA and NA genes from P10 and the six internal genes from WF10 was produced (Table 1).

The role of neuraminidase of H9N2/G1 in relation to human tissue tropism was studied by using two recombinant H9N2 viruses: a H9N2 (rH9N2) containing the HA and NA of G1 and the internal genes from a pandemic H1N1 virus A/California/04/2009 (H1N1pdm), and a comparable virus (rH9N1) with only HA of G1 and the other seven genes from A/California/04/2009.

### *Ex vivo* organ culture and infection

Fresh biopsies of human bronchi and lung from human lung samples were collected from patients undergoing surgical resection of bronchus and lung tissues and infected as previously described^39–41^. Informed consent has been obtained from all subjects and only normal tissues were used for infection. This study was approved by the Institutional Review Board of the University of Hong Kong and Hospital Authority Hong Kong West Cluster (UW 14–119) and all methods involving human tissues were performed in accordance with relevant guidelines and regulations. Samples for *ex vivo* cultures of human bronchus and lung were provided by at least three independent donors. Bronchial and lung tissues were infected with the H9 viruses with a viral titer of 10^6^ 50% tissue culture infectious doses (TCID_50_)/ml, similar to that used previously^39–41^, for 1 h at 37 °C and washed with 5 ml of PBS for three times to remove unbound virus. Mock-inoculated tissues served as negative controls. Viral replication was determined by examining the viral titer from the supernatant cultures collected at 1, 24 and 48 hours post infection (hpi).

### Virus titration assay and area under curve (AUC)

Viral titration assay in MDCK cells was performed as described previously and expressed as TCID_50_/ml^39–41^. Serial dilutions of culture supernatants from infected cultures were titrated in parallel onto confluent 96-well tissue culture plate of MDCK cells plates in quadruplicate and cytopathic effect (CPE) was monitored daily. The end point of viral dilution leading to CPE in 50% of inoculated wells was estimated using the Karber method. The kinetics of thermal inactivation of these H9 viruses over time were also performed at 37 °C by adding 1 ml of virus into 24-well plates with different virus titres (10^4^ and 10^3^ TCID_50_/ml) in the absence of permissive cells. 130 μl of supernatant was collected at 1, 24, and 48 h post incubation to measure viral titer. The replication curves were plotted using the log_10_-transformed viral titers among different viruses at different time points post-infection (Supplementary materials, Figure S1). Since the thermal inactivation curves of these H9 viruses showed virus infectivity decreasing to undetectable levels by 24 hpi (Supplementary materials, Figure S2), the extent of the overall viral replication was assessed with the area-under-curve (AUC) analysis using a trapezoid rule^42^.

### Nuclear magnetic resonance (NMR) spectroscopy

Enzymatic activity of viral NA against 3′-Sialyl-N-acetyllactosamine (3′SLN), 6′-Sialyl-N-acetyllactosamine (6′SLN), N-Acetylneuraminyl-galactose (Neu5Ac-Gal) and N-Glycolylneuraminicyl-galactose (Neu5Gc-Gal) was detected with NMR spectroscopy as detailed previously^43^. All NMR experiments were performed on a Varian 750 MHz spectrometer. H9N2 viruses were purified by ultracentrifugation and fixed in 0.2% paraformaldehyde for 16 hours. ^1^H NMR spectra were obtained every 5 minutes during the 1 h incubation of the virus with glycan substrates at 37 °C. Cleavage of sialic acid (Sia) moieties of the substrates was calculated by the change of chemical shift of the methyl protons within the N-acetamido group.

### Immunohistochemistry

Organ cultures of the respiratory tissue were fixed and paraffin embedded for sectioning and the detection of influenza antigen as described previously^44^. Sections were incubated with HB65 antibody against the nucleoprotein protein for 1 h at room temperature, followed by the addition of biotinylated rabbit anti-mouse antibody. Sections were developed with a Vector NovaRed substrate kit (SK-4800).

### Statistical analysis

Experiments were performed independently at least three times, with different donors. The extent of the overall viral replication of each virus in the experiment time course was represented by the AUC analysis using a trapezoid rule as described^42^. Results shown in the figures are the calculated mean AUC and standard deviation (SD) from such replicate experiments. The AUCs were compared among viruses by one-way ANOVA followed by *Bonferroni* multiple-comparisons test. Differences were considered significant at a *p* < 0.05. The statistical analysis was performed using Graph-Pad Prism 6.0 version for Mac.

### Biosafety and biosecurity

All the experiments that involved the use of the recombinant viruses were conducted in a biosafety level 3 facility at the University of Hong Kong and were risk assessed and approved by the both the Departmental Safety Committee of the School of Public Health, LKS Faculty of Medicine, as well as the Biosafety Committee of the University of Hong Kong. In addition, this study has passed both the biological and biosecurity risk assessment of the University risk assessment for work with infectious agents.

## Electronic supplementary material

Supplementary materials
