# Supplementary material for: Replication of H9 influenza viruses in the human ex vivo respiratory tract, and the influence of neuraminidase on virus release
Source: Sci Rep. 2017 Jul 24;7:6208. doi: 10.1038/s41598-017-05853-5 (PMC5524967; doi:10.1038/s41598-017-05853-5)
Supplement: Supplementary file 1 — Supplementary materials [file 41598_2017_5853_MOESM1_ESM.pdf]

Supplementary materials

Replication of H9 influenza viruses in the human ex vivo respiratory tract, and the influence of neuraminidase on virus release

Renee WY Chan[a,b]# ,Louisa LY Chan[a] ,Chris KP Mok[c] ,Jimmy Lai[c] ,Kin P Tao[a,b], Adebimpe Obadan[d] ,Michael CW Chan[a] ,Daniel R. Perez[d] ,JS Malik Peiris[a] ,John M Nicholls[e]#

School of Public Health, LKS Faculty of Medicine, The University of Hong Kong, Pokfulam, Hong Kong SAR, China [a]; Department of Paediatrics, Faculty of Medicine, The Chinese University of Hong Kong (present address) [b]; HKU-Pasteur Research Pole, The University of Hong Kong [c]; Department of Population Health Poultry Diagnostic and Research Center, College of Veterinary Medicine University of Georgia, Athens, US [d]; Department of Pathology, The University of Hong Kong, Queen Mary Hospital, Pokfulam, Hong Kong SAR, China[e]

#Address correspondence to Renee Wan Yi CHAN and John Malcolm NICHOLLS

				HA (H3 numbering)								NA (N2 numbering)							M1	M2		NS	PB1		PB2		
				Receptor binding site							Cleavage site							Stalk deletions									
Strain name	Subtype	Abbreviation		183	189	190	225	226	227	228		114	247	257	344	391	466		15	28	55	227-229	13-14	667	271	627	701
A/Shanghai/2/2013	H7N9	Sh2	WT	H	A	E	G	L	S	G	PKGR	V	T	I	N	I	Y	Not comparable	I	V	F	KPE	PV	I	T	K	D
A/Quail/Hong Kong/G1/1997	H9N2	G1	WT		T		D		Q		PARSSR	I	S		R	Q	F	38-39				EPE	PA			E	
A/Duck/Hong Kong/Y280/1997	H9N2	Y280	WT	N		T	G											63-65					PV				
A/Duck/Shantou/2030/2001	H9N1	Dk/2030	WT	H		E		Q			PAASDR		N		N		N/A	None	V	I	L	ESE	PA		N/A		
A/Chicken/Hong Kong/SSP117W/2009	H9N2	Ck/SSP	WT	N		A		L			PARSTR		S		R		F	None	I	V	F	GPE	PV		T		
A/Chicken/Hong Kong/YU341/2008	H9N2	Ck/YU341	WT								PSRSSR							None									
A/Chicken/Hong Kong/NT449/2007	H9N2	Ck/NT449	WT	H		D		Q			PARSNR				I		L	None		A		EPE	PA				
A/Hong Kong/1073/1999	H9N2	1073	WT			E		L			PARSSR				R		F	38-39		V							
A/Hong Kong /2108/2003	H9N2	2108	WT	N		V											L	None				GPK			A		
A/Hong Kong/226995/2008	H9N2	226995	WT			A					PSRSSR						F	None	N/A								
A/Hong Kong/464419/2009	H9N2	464419	WT	H		D		Q			PARSNR						L	None	I	A	F	GPE	PA	I	T	E	N
A/Hong Kong/464419/2009 HA-D190E	H9N2	D190E	RG			E												None							T		
A/California/04/2009	H1N1pdm	Ca04	WT		A	D	D		E		N/A	V	N		N	F	F	Not comparable		I		GTE			A		D
G1 HANA: 6 × A/California/04/2009 internal genes	H9N2	rgH9N2	RG		T	E		L	Q		PARSSR	I	S		R	Q		38-39									
G1 HA: 7 × A/California/04/2009 genes	H9N1	rgH9N1	RG									V	N		N	F		Not comparable									
A/guinea fowl/Hong Kong/WF10/1999	H9N2	WF10	WT				G					N/A															
A/Memphis/14/1998	H3N2	M98	WT	N/A																							
A/Netherlands/602/2009	H1N1pdm	/	WT	H	A	D	D	Q	E	G	N/A	V	N	I	N	F	F	Not comparable	I	I	F	GTE	PA	I	A	E	D
WF10 HA: 7 × A/Netherlands/602/2009 genes	H9N1	1WF10	RG		T	E	G	L	Q		PARSSR																
WF10 HANA: 6 × A/Netherlands/602/2009 internal genes	H9N2	2WF10	RG									N/A															
WF10 HANA: 6 × M98 internal genes	H9N2	2WF10:6M98	RG																N/A								
A/Ferret/Maryland/P10_UMD/2008	H9N2	P10	AD		A							I	S	I	R	Q	F	38-39	V	V	F	RSK	PA	I	A	K	D
P10 HA: 7 × A/Netherlands/602/2009 genes	H9N1	1P10	RG									V	N		N	F		Not comparable	I	I	F	GTE				E	
P10 HANA: 6 × A/Netherlands/602/2009 internal genes	H9N2	2P10	RG									I	S		R	Q		38-39									
P10 HANA: 6 × WF10 internal genes	H9N2	2P10:6WF10	RG									N/A															

Table S1. A comparison of the amino acid sequences of HA, NA, M, NS, PB1 and PB2 protein in the H9 viruses studied. Key: WT, wild type; RG, reverse genetic constructed; AD, ferret-adapted.

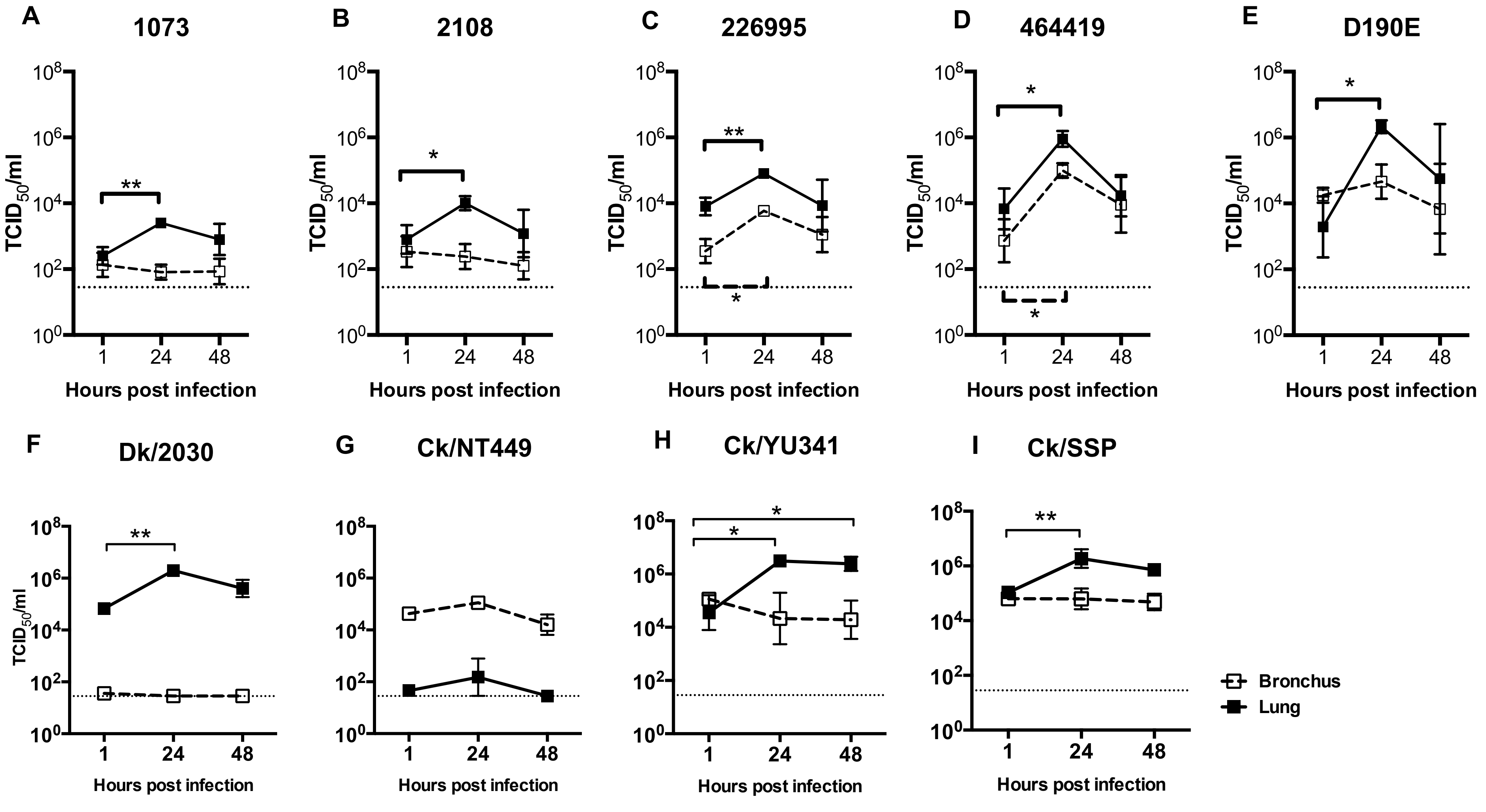

Figure S1. Virus replication kinetics in the human bronchus and lung after the infection of (A to E) human and (F and I) avian H9 viruses. Viral replication kinetics in *ex vivo* cultures of bronchi (open square) and lung (dark square) biopsies infected with 10^6 $TCID_{50}$/ml of virus at 37°C. The chart showed the mean and the SEM of the log_{10} transformed virus titer pooled from at least three independent experiments. Asterisks indicated statistical significant increase in viral yield when compare to 1 hpi. *: $p < 0.05$; **: $p < 0.01$; ***: $p < 0.005$.

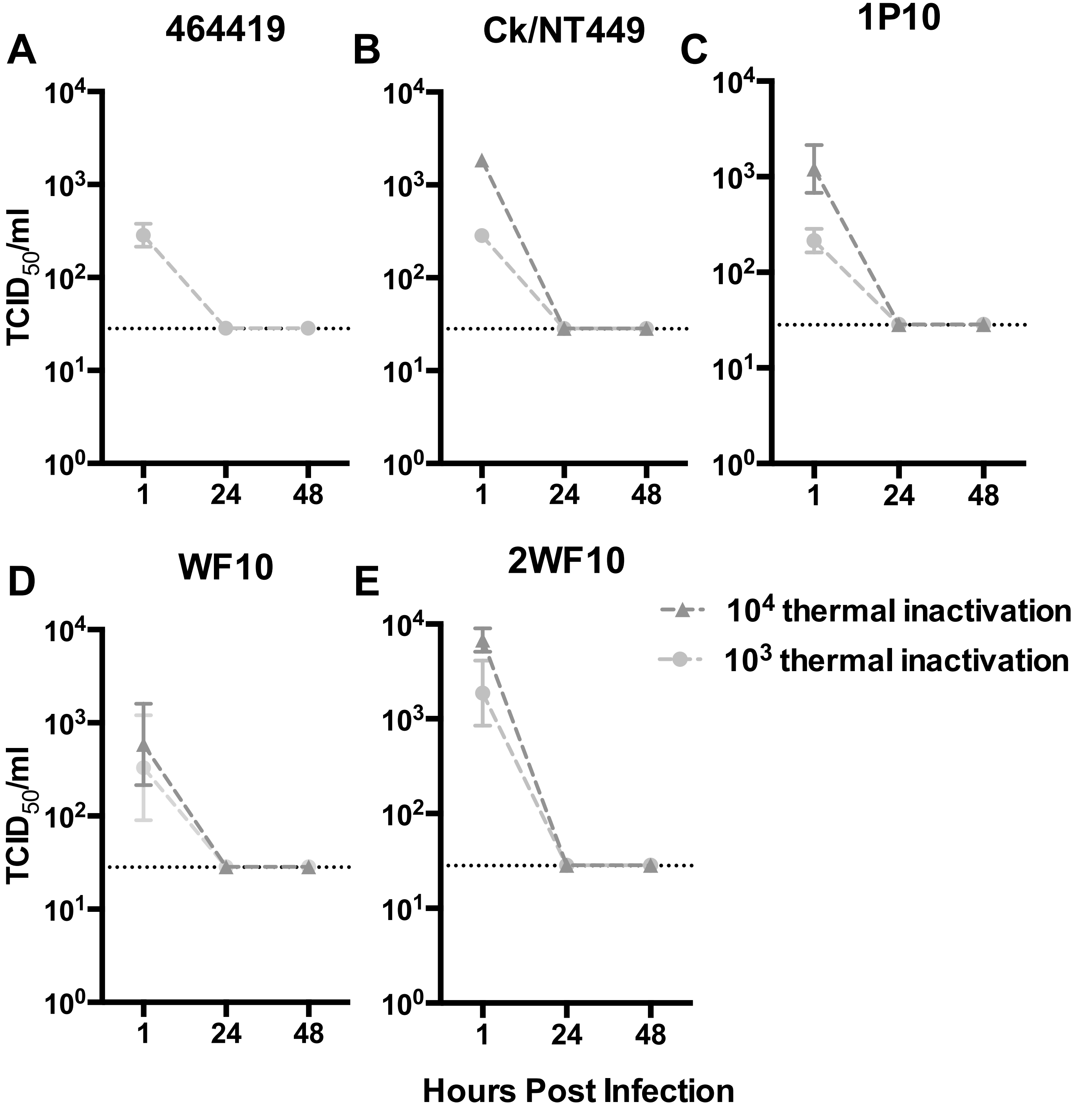

Figure S2. Representative thermal inactivation of H9 viruses at 37°C from 1 to 24 hpi, human virus (A) 464419, avian virus (B) NT449 and recombinant viruses (C) 1P10, (D) WF10, and (E) 2WF10. H9 viruses were all inactivated at 24 h post incubation, irrespective of initial concentration (10^4, grey triangle and 10^3, grey circle $TCID_{50}$/ml). The horizontal dotted line denotes the limit of detection in the $TCID_{50}$ assay.

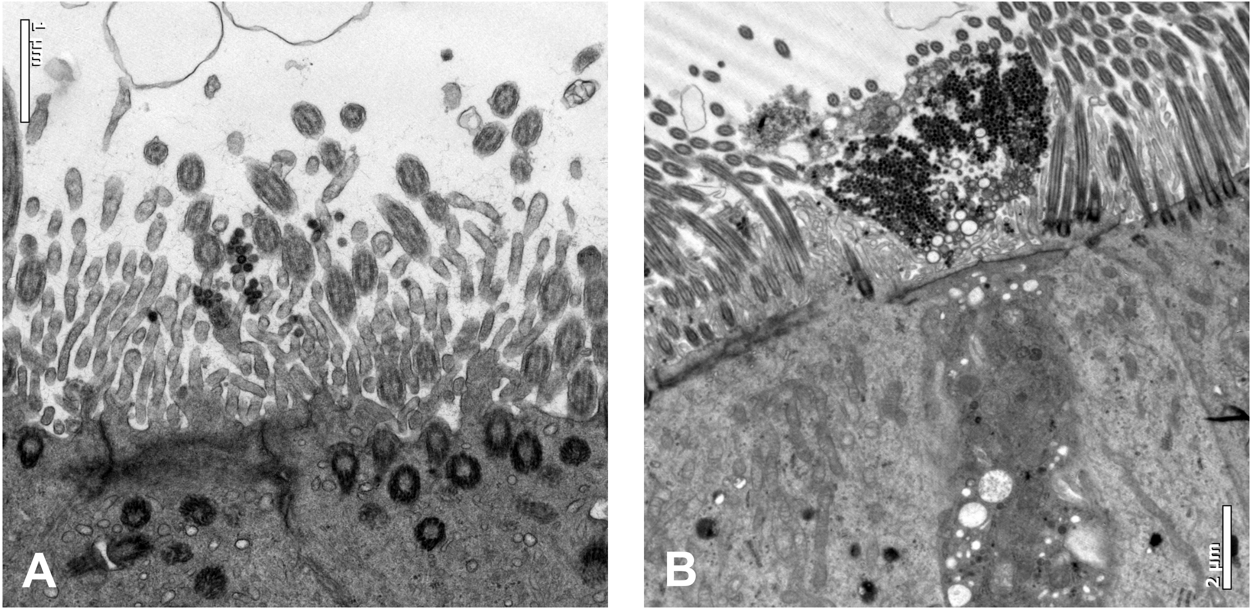

Figure S3. Transmission microscopy of a human *ex vivo* bronchial culture infected with (A) H9N2 D190E and (B) 226995 and fixed by glutaraldehyde at 24 hours followed by post fixation in osmium tetroxide and resin embedding, showing numerous viral particles on the surface.
